# Comments on Scheffler *et al*. Cytotoxic Evaluation of E-Liquid Aerosol using Different Lung Derived Cell Models. *Int. J. Environ. Res. Public Health*, 2015, *12*, 12466-12474

**DOI:** 10.3390/ijerph13010108

**Published:** 2016-01-06

**Authors:** Riccardo Polosa, Massimo Caruso, Francesca Guarino

**Affiliations:** 1 Dipartimento di Medicina Clinica e Sperimentale, Università di Catania, Via Palermo 636, 95121 Catania, Italy; mascaru@unict.it; 2 Dipartimento di Scienze Biomediche e Biotecnologiche, V.le A. Doria 6, 95125 Catania, Italy; fguarin@unict.it

**Keywords:** vapour emission, electronic cigarette, cytotoxicity, tobacco product directive

## Abstract

There is merit in considering a simple toxicological screening method that evaluates the total cytotoxic potential of e-liquids or electronic cigarettes (ECs) aerosol emissions in one single testing. However, there is growing confusion, with several researchers endorsing their personal solution to the problem. Here, we discuss as an example the recent paper by Scheffler and colleagues, in which the authors suggest that more relevant and well differentiated cell lines from human airways could be the most suitable candidates for toxicological evaluation of ECs aerosol emissions. We advance recommendations for validated protocols and advocate for an international coordinated effort aimed at establishing consensus on methodology.

According to the tobacco product directive (TPD) of the European Union, electronic cigarettes (ECs) will be regulated from May 2016 [1]. In particular, the TPD obliges manufacturers and importers of ECs and refill containers to submit a pre-marketing notification to the competent authorities of the Member States of any such products, which they intend to place on the market. The notification shall contain some information, including toxicological data regarding aerosol emissions of the product. It requires the manufacturers to submit what toxicological data they have (e.g., a compilation of the literature data publicly available), but it does not require them to do and submit with the notification any specific toxicological testing, as there is no explicit mention in either the TPD or in the draft of Implementing Act. However, the pre-marketing notification can be supplemented by any toxicological testing with the product the manufacturers may have gathered for themselves.

In relation to this, it is worth noting that there is merit in considering a simple toxicological screening method that evaluates the total cytotoxic potential of e-liquids or ECs aerosol emissions in one single testing, rather than presenting a long list of toxicological risk assessment of several dozens of chemicals tested in isolation. An additional advantage is that toxicological findings from such *in vitro* cellular cytotoxicity system may also detect toxicological potential attributable to unknown contaminants/by-products in the ECs emissions.

Although it should not be too difficult to put this in practice, there is growing confusion with several researchers endorsing their personal solution to the problem. As an example, we discuss the recent paper by Scheffler and colleagues [2]. The authors try to address the need for suitable cytotoxicity models to test e-liquids or ECs aerosol emissions, by suggesting that their in-house immortalized human bronchial epithelial cell line (*i.e.*, CL-1548) would be the most suitable candidate for toxicological evaluation.

Their working hypothesis is that it is important to consider the anatomical site of primary impact of aerosols (*i.e.*, the conducting zone of the respiratory tract) in order to establish a more relevant cell culture model for a toxicological evaluation of ECs emissions. Based on the assumption that the most of these emissions impact on the respiratory tract (and not on the alveolar lining), they conclude that the human bronchial epithelial cell culture is the most suitable model and propose their in-house immortalized human bronchial cell line as a candidate (*i.e.*, CL-1548). The main problem with this approach is that fully characterized human bronchial epithelial cell lines are already available from ATCC (e.g., BEAS-2B, 16HBE) and generally used for regulatory purposes by FDA [3,4] and that cell differentiation is not an essential requirement for cytotoxicity testing. 

The authors compared cell viability of ECs aerosol emission to that of cigarette smoke (positive control) and clean air (negative control) 24-h post exposure of normal human bronchial epithelial (NHBE) cells, immortalized human bronchial epithelial (CL-1548) cells, and adenocarcinoma human alveolar basal epithelial (A549) cells. After 24-h incubation with aerosol emission, cell viability was reduced in CL-1548 much more than A549 and less than NHBE and based on this observation the authors conclude that it is best to use CL-1548 for testing of ECs aerosol emissions by virtue of its heightened cytotoxic sensitivity. The sensitivity of a cell line is a relative concept and it is not surprising that different cell types expressing different grade of differentiation may exhibit different sensitivity. Therefore, conclusions about the appropriateness of a cell line compared to another, as the most “suitable” candidate for toxicological evaluation, requires justification beyond simplistic considerations about the anatomical site of primary impact of aerosols. What if after exposure ECs aerosol emissions, cell viability was not reduced in alternative bronchial epithelial cell lines (e.g., BEAS-2B, 16HBE)? These cell lines should be included for cross checking purposes and to support these authors working hypothesis. Nonetheless, cell differentiation is not an essential requirement for regulatory cytotoxicity studies, it may be a valid scientific approach to when addressing other aspect concerning bronchial epithelial health (e.g., reduction in cilia beating frequency, electrophysiological studies for establishing dysfunctional tight junctions, *etc*.).

It is also unjustified to select a specific cell lines just because generates a predefined response. It is very common among researchers to go for those cell lines that generate responses of interest. Thus, appropriateness of a cell line compared to another as the most “suitable” candidate can be also dictated by evidence of positive responses and not by rational choices.

When analyzing positive responses in term of cell toxicity, Scheffler and coll. [2] paid great attention to the anatomical site of primary impact of aerosols, but failed to recognize that the aerosol and smoke generation protocols are the most important factor that influences cytotoxicity. For this reason, it is more important to establish the correct exposure protocols (time, dose) in relation to the culture model utilized. There is no justification for exposing cell cultures to 200 puffs for ECs and only to 60 puffs for conventional cigarettes. This choice is arbitrary and will introduce bias when comparing cytotoxicity between ECs aerosol emissions and tobacco smoke. These methodological problems often arise when not using validated protocols.

In conclusion, when assessing potential cytotoxicity effects of ECs aerosol emissions, it is mandatory to compare them with those resulting from the exposure of cigarette smoke. In the absence of clearly defined ECs aerosol generation methods and exposure protocols, it is recommended to perform an ISO 10993-5 [5] study on a human bronchial epithelial cell lines available from ATCC (e.g., BEAS-2B, 16HBE). The ISO 10993-5 protocol has pre-determined toxicity end-points (*i.e.*, <70% cell survival), defines the level of exposure (extract of 1% concentration) and is used for approval of medical devices or products. It is critical that future evaluation of the harm potential of ECs aerosol emissions gets away from the controversial toxicological debate that has been generated in recent laboratories studies because of experimental protocols that do not mimic realistic condition of use [6]. Last but not least, given the concerning lack of uniformity in methods used to generate ECs aerosol emissions [7], it is crucial to set up an internationally coordinated effort aimed at establishing technical consensus if we wish to advance science and better inform regulators.
